# Unique chromoplast organisation and carotenoid gene expression in carotenoid-rich carrot callus

**DOI:** 10.1007/s00425-018-2988-5

**Published:** 2018-08-21

**Authors:** Tomasz Oleszkiewicz, Magdalena Klimek-Chodacka, Anna Milewska-Hendel, Maciej Zubko, Danuta Stróż, Ewa Kurczyńska, Aleksandra Boba, Jan Szopa, Rafal Baranski

**Affiliations:** 1 Institute of Plant Biology and Biotechnology, Faculty of Biotechnology and Horticulture, University of Agriculture in Krakow, AL. 29 Listopada 54, 31-425 Kraków, Poland; 2 Department of Cell Biology, Faculty of Biology and Environmental Protection, University of Silesia in Katowice, Jagiellońska 28, 40-032 Katowice, Poland; 3 Institute of Materials Science, University of Silesia in Katowice, 75 Pułku Piechoty 1a, 41-500 Chorzow, Poland; 4 Department of Genetic Biochemistry, Faculty of Biotechnology, University of Wroclaw, Przybyszewskiego 63/77, 51-148 Wrocław, Poland; 5 Department of Genetics, Plant Breeding and Seed Production, Wroclaw University of Environmental and Life Sciences, Pl. Grunwaldzki 24A, 50-363 Wrocław, Poland

**Keywords:** Callus tissue in vitro, Carotenoid biosynthesis pathway, Chromoplast biogenesis, Chromoplast ultrastructure, Transcript level, Ultra performance liquid chromatography (UPLC)

## Abstract

The new model orange callus line, similar to carrot root, was rich in carotenoids due to altered expression of some carotenogenesis-associated genes and possessed unique diversity of chromoplast ultrastructure.

Callus induced from carrot root segments cultured in vitro is usually pale yellow (p-y) and poor in carotenoids. A unique, non-engineered callus line of dark orange (d-o) colour was developed in this work. The content of carotenoid pigments in d-o callus was at the same level as in an orange carrot storage root and nine-fold higher than in p-y callus. Carotenoids accumulated mainly in abundant crystalline chromoplasts that are also common in carrot root but not in p-y callus. Using transmission electron microscopy, other types of chromoplasts were also found in d-o callus, including membranous chromoplasts rarely identified in plants and not observed in carrot root until now. At the transcriptional level, most carotenogenesis-associated genes were upregulated in d-o callus in comparison to p-y callus, but their expression was downregulated or unchanged when compared to root tissue. Two pathway steps were critical and could explain the massive carotenoid accumulation in this tissue. The geranylgeranyl diphosphate synthase gene involved in the biosynthesis of carotenoid precursors was highly expressed, while the β-carotene hydroxylase gene involved in β-carotene conversion to downstream xanthophylls was highly repressed. Additionally, paralogues of these genes and phytoene synthase were differentially expressed, indicating their tissue-specific roles in carotenoid biosynthesis and metabolism. The established system may serve as a novel model for elucidating plastid biogenesis that coincides with carotenogenesis.

**Electronic supplementary material:**

The online version of this article (10.1007/s00425-018-2988-5) contains supplementary material, which is available to authorized users.

## Introduction

Carotenoids are essential in plant development and growth as they are common components of cell photosystems, but in some species they are also sequestered in photosynthetically inactive tissues (Sun et al. 2018). Their presence in human and animal diet is critical as some of them are provitamin A precursors while others are important in age-related dysfunctions such as macular degeneration, and have also antioxidant properties (Milani et al. 2017). The high significance of these compounds boosted research using model plants, and stimulated programmes aimed at the enhancement of crop plants by altering carotenoid content and composition (Giuliano 2017).

Accumulation of carotenoids in plants is determined by the rate of biosynthesis and subsequent degradation carried out in plastids (Cazzonelli 2011; Schaub et al. 2018), and the pathway of carotenoid metabolism in plants has been thoroughly described (Rodriguez-Concepcion 2010; Ruiz-Sola and Rodríguez-Concepción 2012). Regulation of carotenoid biosynthesis and accumulation is complex and has not been fully elucidated. It involves a cross-talk between the 2-C-methyl-d-erythritol 4-phosphate (MEP) pathway delivering substrates for carotenogenesis and the carotenoid pathway delivering signal molecules for the feedback response. Environmental factors regulate carotenoid biosynthesis that is then manifested in qualitative and quantitative changes of these metabolites (Nisar et al. 2015). Sunlight is the primary modulator affecting the transcription level of key genes such as phytoene synthase (*PSY*) through phytochrome and cytochrome receptors transducing the light signal to induce or repress gene expression (Llorente et al. 2017). Developmental regulation is common and clearly observed in ripening fruits while changing their colour due to altered pigment accumulation accompanying chloroplast degradation and chromoplast development (Cazzonelli and Pogson 2010; Sun et al. 2018). Direct evidence that carotenoid accumulation is regulated by genetic determinants related to plastid biogenesis was found in cauliflower (Lu et al. 2006), but plastid differentiation is additionally affected by environmental stimuli (Li and Yuan 2013). Plastids may undergo transition from initial proplastids to non-green leucoplasts and amyloplasts, and green chloroplasts. In non-green tissues, chromoplasts originating from chloroplasts or non-green plastids have the capacity to sequester carotenoids and store them safely for a long period (Egea et al. 2010). Chromoplasts are the primary location of carotenogenesis, with most enzymes associated with membranes, although some of them remain active in stroma or are associated with plastid sub-structures (Shumskaya and Wurtzel 2013; van Wijk and Kessler 2017).

Carrot (*Daucus carota* L.) is a vegetable accumulating high amounts of carotenoids in its storage root; thus it is one of a few plant species synthesizing these pigments in an underground organ. The ability of carrots to accumulate large quantities of predominantly provitamin A carotenoids has been intriguing for decades, but its mechanism still remains not fully elucidated although structural genes and quantitative trait loci (QTL) were identified and mapped (Just et al. 2007; Cavagnaro et al. 2011). The activity of carotenoid genes changes during carrot root development, but increased transcript levels only partially explain massive carotenoid sequestration (Clotault et al. 2008). Recently, a mutant gene outside the carotenoid biosynthesis pathway and related to photomorphogenesis has been proposed as a putative regulator essential for carotenoid accumulation in the orange storage root (Iorizzo et al. 2016). The lack of a direct correlation between transcript and carotenoid levels indicates the essential role of plastid biogenesis in carotenoid biosynthesis, accumulation and storage. This was also shown in dark-grown and de-etiolated carrot roots where carotenoid level was coordinated with light control and chromoplast development (Fuentes et al. 2012; Rodriguez-Concepcion and Stange 2013). Typical chromoplasts in an intact carrot root contain mainly crystalline sub-structures resulting from overproduction of carotenes which are then stored in lipoprotein sheets, and their unidirectional growth causes the formation of needle-like, broad ribbon-like, and tube-like structures that expand along the plastid envelope (Frey-Wyssling and Schwegler 1965). Recent studies confirmed that carrot chromoplasts contain carotenoids in a solid-crystalline physical state (Schweiggert et al. 2012). The crystals are composed predominantly of β-carotene and may also contain α-carotene as identified using spectroscopy (Roman et al. 2015; Rygula et al. 2018). Globular chromoplasts containing lipid-dissolved carotenoids in plastoglobuli were also found in carrot root cells, but they were small in size and number (Kim et al. 2010). A direct relationship between chromoplast type and carotenoid content and composition has also been observed in other species, confirming that carotenogenesis is a species-specific process (Kilcrease et al. 2013; Schweiggert and Carle 2017).

Carrot callus cultures have been widely used in research for almost 80 years, which began when carrot became one of the first species reported to develop a callus in vitro and was used for the establishment of a cell suspension culture to prove the concept of plant cell totipotency (Ikeuchi et al. 2013). Callus cells’ ability to differentiate into different types of cells makes this tissue a feasible model for basic research at a cellular level. Carrot callus can be easily propagated and exposed to different stress factors. It can also serve as a source of released cells and protoplasts, and is amenable to genetic transformation (Baranski 2008). These features make carrot callus a convenient laboratory-based model system that has been recently demonstrated for precise gene editing purposes (Klimek-Chodacka et al. 2018). Genetically modified callus was also valuable for studying physiological processes and their molecular control in other species, including carotenoid biosynthesis, regulation and accumulation (Kim et al. 2013; Schaub et al. 2018).

Carrot callus developing from cambium of root explants is poor in carotenoids and has a pale yellow colour, even when discs of orange root are cultured in vitro (Baranska et al. 2006; Baranski et al. 2006). Recently, we have established a pair of stable callus lines derived from the same orange carrot root that accumulate either low or high amounts of carotenoids, the latter being essentially similar to that in the carrot root. These lines can serve as a unique model for plant carotenoid research, and both chemical and structural differences of carotenoid crystals have already been demonstrated in this orange callus by applying simultaneously three non-destructive and complementary techniques: Raman imaging, atomic force microscopy (AFM) and scanning near-field optical microscopy (SNOM). Moreover, SNOM observations of intact cells in carotenoid-rich callus have indicated the presence of carotenoid-containing subcellular structures that resembled not only crystalline but also membranous and tubular chromoplasts (Rygula et al. 2018).

The results presented in this work demonstrate unique histology, ultrastructure and chromoplast diversity accompanying high carotenoid contents of the established carotenoid-rich carrot callus. A distinct expression pattern of carotenoid-associated genes is shown, including variant roles of some paralogues. The results provide evidence that this cell system may constitute a novel model for basic research on carotenogenesis and chromoplast biogenesis.

## Materials and methods

### Plant materials

**Fig. 1:**
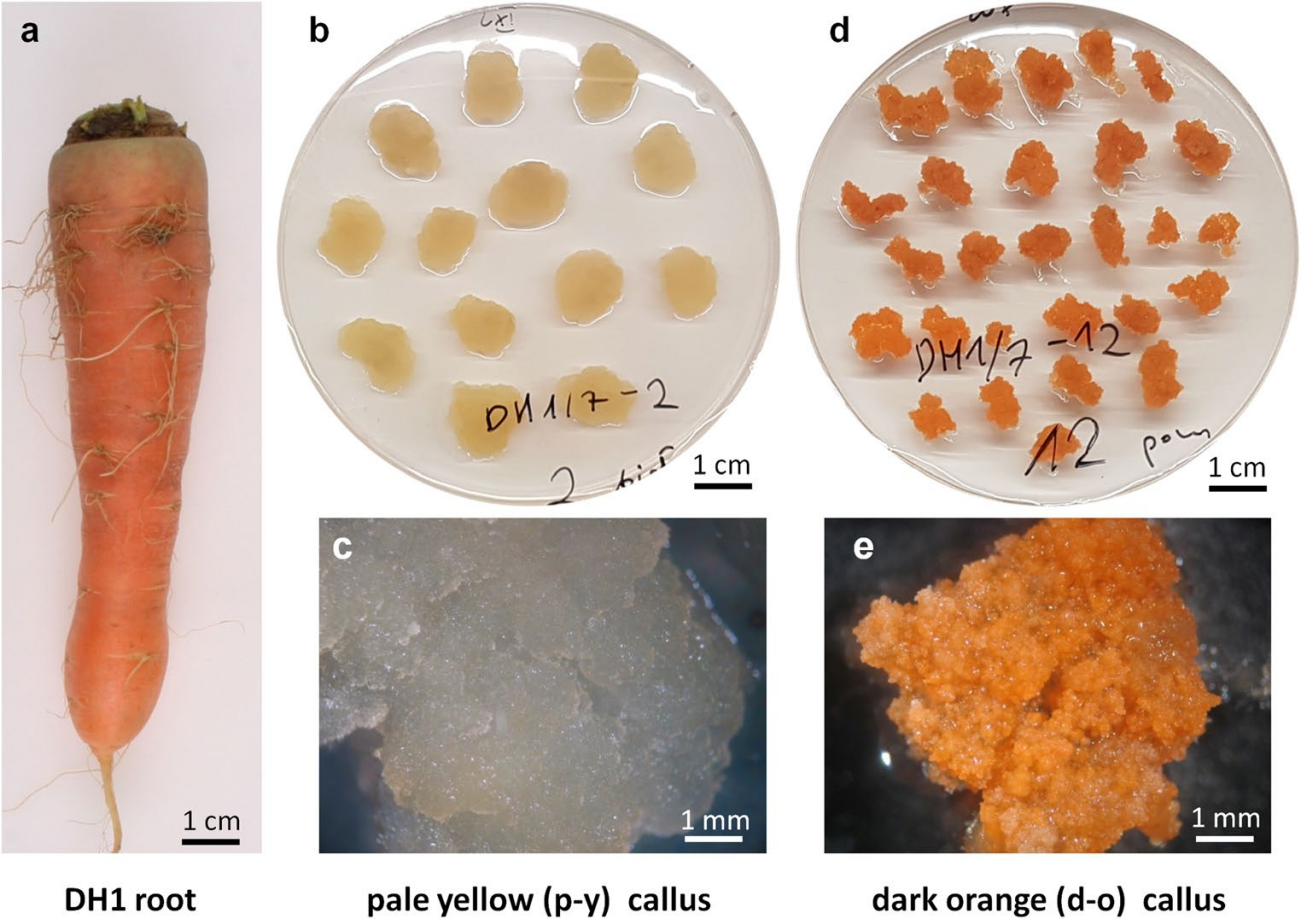
Root of DH1 carrot line used for callus induction (**a**). Pale yellow (p-y) callus developed from cambium of root discs and cultured on BI mineral medium in vitro (**b**), and observed under higher magnification (**c**). Dark orange (d-o) callus line established from p-y callus by visual selection and subsequent subculture (**d**), and observed under higher magnification (**e**)

Orange roots of the DH1 doubled haploid carrot (*Daucus carota* L. subsp. *sativus* Hoffm.) line (Fig. 1a) were surface sterilized and cut transversally into 5 mm thick discs, which were then cultured on BI medium (Gamborg B5 mineral medium with vitamins; Duchefa, Haarlem, The Netherlands) supplemented with 1 mg/L 2,4-dichlorophenoxyacetic acid, 0.0215 mg/L kinetin and 30 g/L sucrose, pH 5.8, solidified with 2.7% Phytagel, at 26 °C in the dark. After 2 months, pale yellow (p-y) callus developing along the cambium was transferred to BI medium and cultured in the same conditions, producing stable lines (Fig. 1b, c). Within consecutive subculture periods, small orange-coloured cell foci appeared in the p-y callus line. Orange cell clumps were subsequently transferred to BI medium until a dark orange (d-o) callus line was established (Fig. 1d, e). Visual selection of orange cells was necessary to eliminate non-pigmented cells, which otherwise overgrew the tissue, and to obtain a stable orange callus line. Callus transfer to fresh medium was done every 4 weeks.

### Isolation of terpenoids and UPLC analysis

Tissue was ground in liquid nitrogen and lyophilized in the dark. Aliquots of 50 mg were extracted using successively methanol, acetone and petroleum, and ultra performance liquid chromatography (UPLC) was then used to detect terpenoids as described in detail by Boba et al. (2018).

### Abscisic acid isolation and quantification

Abscisic acid (ABA) was extracted from 200 mg of fresh tissue ground in liquid nitrogen according to Boba et al. (2018). ABA content was determined spectrophotometrically at *λ* = 450 nm in a Varioscan Microplate Reader (Thermo Fisher Scientific) using the Abscisic Acid Immunoassay Detection Kit (Sigma-Aldrich) according to the producer’s protocol.

### Microscopic identification of carotenoid crystals

A fresh callus sample was placed on a microscopic slide in a drop of demineralised water, covered with a cover glass and observed without any processing using the Axiovert S100 (Carl Zeiss) bright-field microscope and the Axioskop 40 (Carl Zeiss) polarizing microscope equipped with the MOTICAM580 5.0 MP digital camera with the corresponding software (Nikon).

### Histological observation of cells

Samples for histological analyses were collected at various callus areas and fixed in a mixture of 4% (w/v) paraformaldehyde (PFA; Polysciences) and 1% (v/v) glutaraldehyde (GA; Sigma-Aldrich) in phosphate buffered saline (PBS, pH 7.0) at 4 °C for 24 h. Then samples were washed in PBS, dehydrated in a graded ethanol series and infiltrated in LR White resin (Polysciences). Later, polymerization samples were cut into 1.5 µm thick sections using the Leica EM UC6 ultramicrotome, mounted on microscope slides, stained with aqueous solutions of 0.05% Toluidine Blue O (TBO; Sigma-Aldrich) for 10 min, rinsed with distilled water, covered with a cover glass and analyzed with the Nikon Eclipse Ni-U bright-field microscope equipped with the Nikon Digital DS-Fi1-U3 camera with corresponding software. At least three samples were collected and analyzed from each callus line.

### Transmission electron microscopy (TEM)

Samples for TEM were fixed in 2.5% glutaraldehyde and 2.5% paraformaldehyde in 0.05 M cacodylate buffer (Sigma-Aldrich) (pH 7.2), kept at 4 °C for 24 h, post-fixed in 1% osmium tetroxide (Sigma-Aldrich) in distilled water at 4 °C overnight, dehydrated in a graded series of ethanol and gradually embedded in Epon resin (Poly/Bed 812; Polysciences) according to a method described previously (Milewska-Hendel et al. 2017). Ultrathin 70-nm thick sections were obtained with the Leica EM UC6 ultramicrotome and collected onto carbon-coated copper grids (300 mesh, Electron Microscopy Science, Hatfield, PA, USA). Grids with sections were stained with a saturated solution of uranyl acetate (Polysciences) in 50% ethanol for 15 min and 0.04% lead citrate agents (Sigma-Aldrich) for 10 min and analyzed in a Jeol JEM-3010 high resolution electron microscope (HRTEM) (300 kV) equipped with an EDS spectrometer and a 2 k × 2 k Orius 833 SC200D CCD camera (Gatan, Pleasanton, CA, USA). TEM images were taken for at least three samples from each callus line.

Significant differences in the mean number of plastids or plastoglobuli between two callus tissues were determined using a *t* test. Frequencies of chromoplasts containing plastoglobuli were compared using a test for significant differences between two proportions.

### Gene expression

Tissue samples were frozen in liquid nitrogen and ground in a mortar. Total RNA was isolated using the Direct-zol RNA MiniPrep Plus kit with TRI Reagent (Zymo Research, Irvine, CA, USA) and followed by DNase I treatment (Thermo Fisher Scientific). Lack of DNA contamination was verified by PCR and qPCR on the RT control. cDNA was synthesized from 1 µg of RNA using the iScript cDNA Synthesis Kit (Bio-Rad). Real-time quantitative PCR (qPCR) was conducted using the StepOnePlus (Applied Biosystems) thermocycler with the Maxima SYBR Green/ROX qPCR Master Mix (Thermo Fisher Scientific). Primers were validated for single product specificity and their effectiveness to range between 90 and 105% (Electronic Suppl. Table S1). qPCR thermal cycling conditions were: initial denaturation at 95 °C for 10 min, 40 cycles of 95 °C for 15 s and 55 °C for 60 s. Amplification was followed by melt curve analysis to verify single product amplification. Normalization was done to the expression of the actin gene. qPCR reactions were done in at least three biological replications. Relative gene expression was calculated using the REST 2009 (Qiagen) software.

## Results

### Carotenoid content in orange callus is similar as in carrot root

**Fig. 2:**
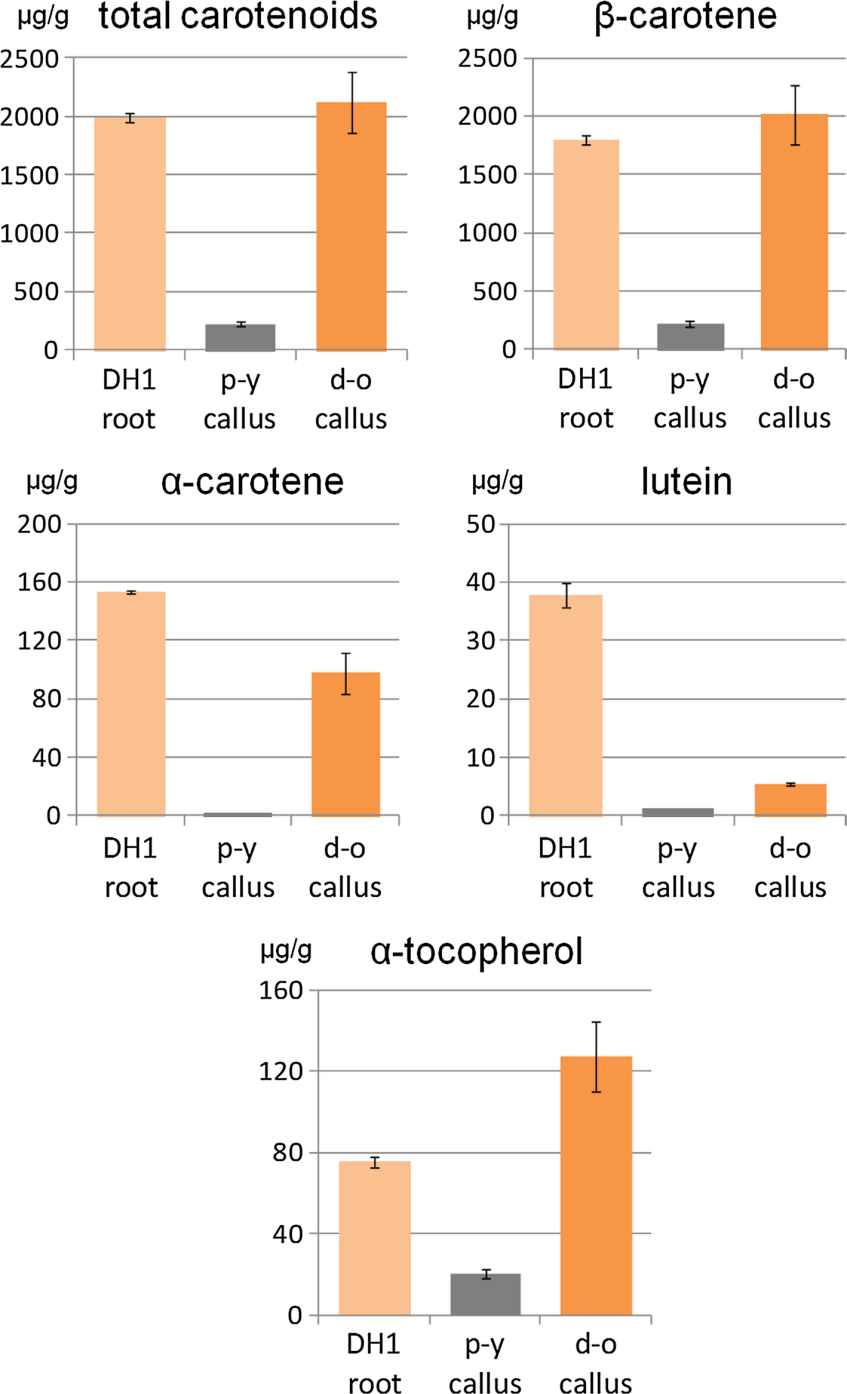
Carotenoid and α-tocopherol contents in roots of the DH1 carrot line and in callus derived from them. p-y—pale yellow callus developed from cambium of DH1 root disc incubated on BI medium in vitro, d-o—dark orange callus selected from p-y callus. Means with standard errors per g tissue dry weight; *n* = 8 (roots), *n* = 5 (p-y callus), *n* = 10 (d-o callus)

Root discs of orange carrot DH1 line (Fig. 1a) were exposed to a mineral medium in vitro, which resulted in the development of pale yellow (p-y) callus tissue along the cambium (Fig. 1b, c), as expected. However, during subsequent subcultures of this tissue, orange-coloured cell foci appeared and they were separated from the host tissue for further growth (Fig. 1c, d). In consequence, a pair of p-y callus and dark orange (d-o) callus lines was established. Carotenoid contents in both p-y and d-o callus lines were determined and compared to the amounts in DH1 roots. The total carotenoid content in the roots was high (1985 µg/g dry weight; DW) and resulted from the presence of three main compounds (Fig. 2). β-carotene predominated and constituted 90% of total carotenoids while α-carotene and lutein constituted only 8 and 2%, respectively. In contrast, p-y callus was poor in carotenoids and contained 8.5-fold lower amounts of carotenoids (232 µg/g DW) than the roots. The contents of α-carotene, lutein, other xanthophylls and phytoene in p-y callus were very low, and thus β-carotene was mainly present (95% of total carotenoids). The selected d-o callus line contained similar amounts of β-carotene (2015 µg/g DW) and total carotenoids (2150 µg/g DW) as the roots (*P* = 0.486 and 0.668, respectively) with β-carotene being the main carotenoid (94%). α-Branch carotenoids accounted for 5% of total carotenoids, and their amounts were lower than in the root by 36 and 85% for α-carotene and lutein, respectively. The comparison of both callus tissues showed that d-o callus contained more total carotenoids (9.3-fold), β-carotene (9.1-fold), α-carotene (48.8-fold), lutein (4.3-fold), other xanthophylls (1.6-fold) and phytoene (3.7-fold) than p-y callus. Additionally, the level of ABA was 3.4-fold higher (*P* < 0.001) in d-o callus (0.41 nmol/g FW) than in p-y callus (0.12 nmol/g FW). The content of α-tocopherol was 3.7-fold lower in p-y callus than in the roots and 6.4-fold lower than in the d-o callus while in d-o callus the content was 1.7-fold higher (*P* = 0.017) than in the roots (Fig. 2).

These results show that the observed orange colour in d-o callus was due to accumulation of carotenoid pigments that were present in similar amounts as in the roots. It was mainly attributed to the presence of β-carotene, slightly elevated amounts of which compensated for the lower α-branch carotenoid contents.

### Differences in p-y callus and d-o callus are prominent at histological and ultrastructural levels

**Fig. 3:**
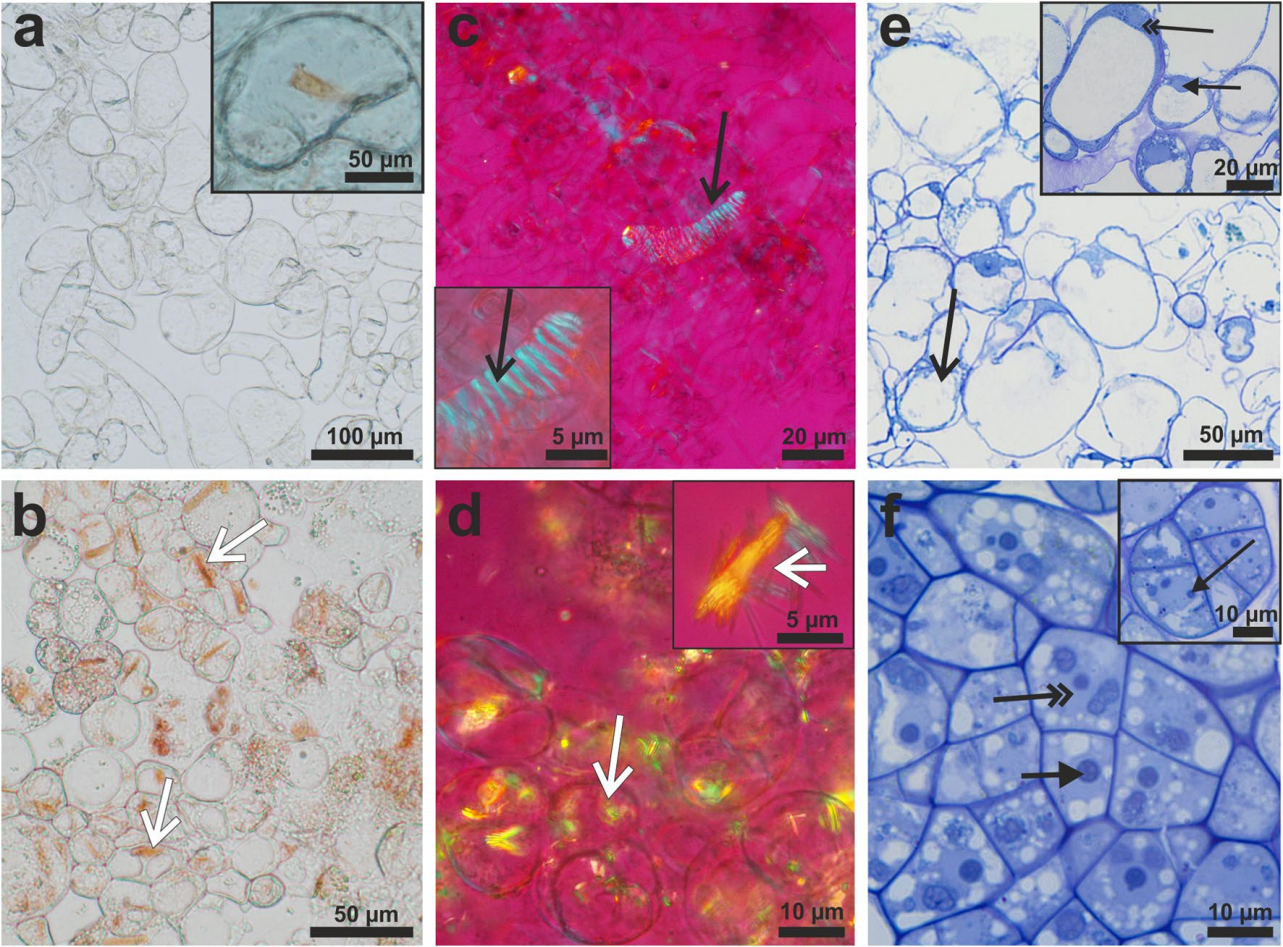
Callus cells in bright-field (**a**, **b**, **e**, **f**) and polarizing microscopy (**c**, **d**). Cells from p-y callus (upper row) almost devoid of carotenoid crystals (**a**, **c**) with rarely observed crystals in single cells (**a** inset). Some callus cells differentiated into tracheary elements (**c** inset: open arrow). In contrast, d-o callus cells (bottom row) were filled with crystals (**b**, **d**, white, open arrows; inset: magnification of crystals released from the cell). Histology of callus tissue stained with toluidine blue O (**e**, **f**). Highly vacuolated and loosely attached cells in p-y callus (**e**). Many cells located at the callus surface presented features resembling those of cell decay (**e**, black, open arrow). Inside p-y callus alive cells with large vacuole, narrow layer of cytoplasm (**e** inset: double arrow) and nucleus (**e** inset: black arrow) were present. Dark orange callus composed of compact arrangement of cells (**f**) having dense cytoplasm, large nucleus (double arrow) with prominent nucleolus (arrow) that indicates their embryogenic character. Some cells from d-o callus were characterized by a dense cytoplasm and a large nucleus with nucleoli (**f** inset: arrow) that indicate their meristematic character

Microscopic observations revealed that cells of p-y callus were almost transparent when observed in a bright field, and that they did not show birefringent crystals when observed using a polarizing microscope (Fig. 3a, c). In some cells, orange, irregular, amorphous and rarely crystalline carotenoids were spotted. In contrast, cells of d-o callus were rich mainly in orange-coloured crystalloid structures of regular shapes (Fig. 3b), which were also clearly visible in polarizing light (Fig. 3d). They occurred in cells either individually or arranged in groups, often closely packed (Fig. 3d).

Both callus tissues differed in their histological and cellular structures. The p-y callus was composed of loosely connected cells, which were large and rounded, with a prominent central vacuole (Fig. 3e). Some cells located deeper in the callus mass also possessed cytoplasm heavily stained with Toluidine Blue O (TBO), and with a clearly visible nucleus (Fig. 3e inset). Many cells were characterized by a large size, prominent vacuole and a narrow strand of cytoplasm in the vicinity of the walls (Fig. 3e), also visible in greater detail at an ultrastructural level, described below. In contrast, d-o callus was composed mainly of embryogenic-like cells characterized by a dense cytoplasm, large nucleus with a prominent nucleolus, small starch grains and a fragmented vacuome (Fig. 3f). Meristematic cells were also present and they were characterized by a dense cytoplasm, large nucleus with at least two nucleoli and small vacuoles. Meristematic and embryogenic cells formed cell aggregates within callus tissue (Fig. 3f).

**Fig. 4:**
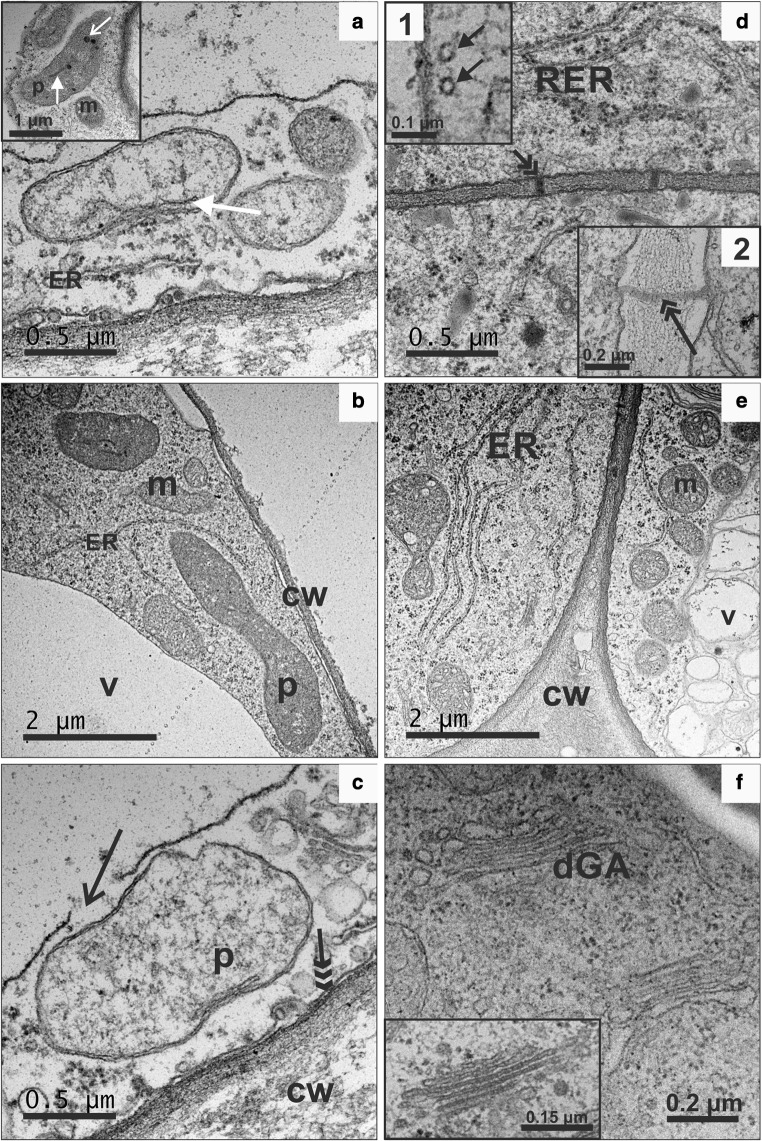
Ultrastructure of cells from p-y (**a-c**) and d-o callus (**d-f**). Cells from p-y calli had electron-lucent cytoplasm with some ER membranes (**a**), mitochondria, a large vacuole, plastids with limited internal membranes and a few plastoglobuli (**a** inset). Cells with a narrow layer of electron-dense cytoplasm filled with numerous ribosomes, ER, plastids, mitochondria and a prominent vacuole were also detected (**b**). In some cells of p-y callus symptoms of cell decay were found (**c**). Cells from d-o callus were characterized by electron-dense cytoplasm (**d**–**f**) with numerous profiles of rough ER (**d**), microtubules, plasmodesmata (PD) [**d,** inset 1: black arrows, higher magnification of microtubules; inset 2: black, double arrow, higher magnification of PD], numerous mitochondria, vacuoles (**e**) and dictyosomes of the Golgi apparatus (**f**, **f** inset). *cw* cell wall, *dGA* dictyosomes of Golgi apparatus, *ER* endoplasmic reticulum, *m* mitochondria, *p* plastids, *RER* rough endoplasmic reticulum, *v* vacuole. Black open arrow, discontinuity of tonoplast; black double arrow, plasmodesmata; black triple arrow, plasma membrane; white arrow, plastid membranes; white open arrow, plastoglobuli

Both types of calli also differed at the ultrastructural level. In p-y callus most cells were highly vacuolated, with one prominent vacuole (Fig. 4a). Cytoplasm was electron-lucent, with only some mitochondria and plastids. The plastid ultrastructure was very simple, with a limited internal membrane system and a few plastoglobuli (Fig. 4a, inset). These plastids were classified as proplastids and were most abundant in p-y callus cells (Fig. 4a; Table 1). Amyloplasts containing large starch grains were less frequent, and plastids with crystal remnants were found occasionally. In some callus regions, cells with dense cytoplasm with a very well-preserved tonoplast, endoplasmic reticulum (ER) profiles and mitochondria were also present (Fig. 4b). Cells showing disruption of the tonoplast membrane continuity (Fig. 4c; open arrow) but with still preserved plasma membrane continuity (Fig. 4c; arrow) were detected. The ultrastructure of d-o callus cells varied. Cells had a dense cytoplasm, numerous mitochondria and high number of Golgi apparatus dictyosomes, indicating intensive exocytosis (Fig. 4d–f). They comprised numerous rough ER (rER) profiles, ribosomes and small vacuoles. Microtubules occurred in the vicinity of the plasma membrane, and plasmodesmata traversing the neighbouring cell walls were also abundant (Fig. 4d). The number of plastids per cell was two-fold higher than in p-y callus and chromoplasts dominated (Table 1). Their number was 2.7-fold higher than the number of amyloplasts and proplastids, while the later were almost three-fold less frequent in d-o callus than in p-y callus. Also the proportion of plastids with plastoglobuli was higher (*P* = 0.032) in d-o callus (0.55) than in p-y callus (0.38), and these plastids contained different average numbers (*P* = 0.026) of plastoglobuli, 3.9 and 2.5, respectively.

**Table 1:** Number of plastids in p-y and d-o callus

Plastid type	p-y callus	d-o callus	*P* ^a^
Proplastids	38.0^b^	13.3	0.002
Amyloplasts	9.7	16.3	0.022
Chromoplasts	3.0	78.7	< 0.001
Crystalline	3.0	43.3	< 0.001
Globular	0.0	16.3	**–**
Membranous	0.0	9.3	**–**
Tubular	0.0	9.7	**–**

^a^*t* test significance level for difference between means

^b^Mean values from three replications of 40 cells each

### Orange callus cells contain various chromoplast types

**Fig. 5:**
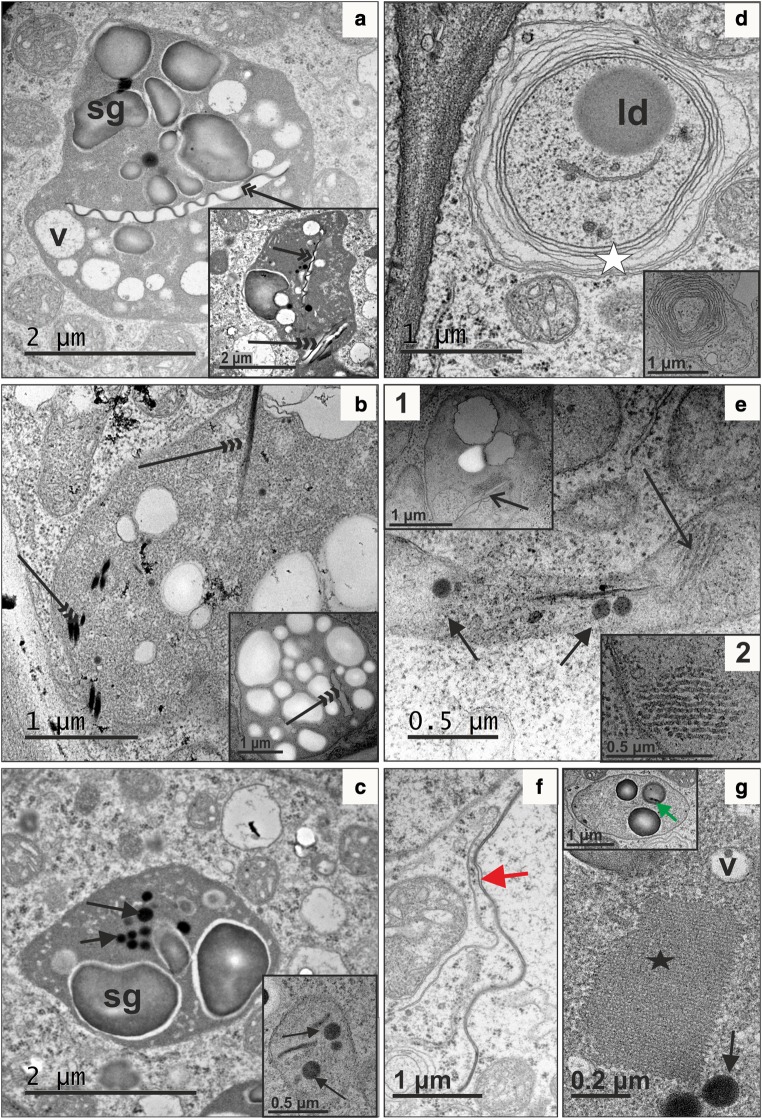
Ultrastructural diversity of chromoplast types in d-o callus: crystalline (**a**, **b**), globular (**c**), membranous (**d**) and tubular (**e, e** inset 2: transverse section). Chromoplast membranes devoid of crystals were frequently quite long and extended outward from the chromoplast membrane (**f**, red arrow). Phytoferritin crystals were occasionally observed (**g**, black star); also large plastoglobuli were detected and in some of them crystal remnants were present (**g** inset: green arrow). Arrow, plastoglobuli; open arrow, tubules inside a chromoplast; double arrow, membrane remnants after removal of carotene crystals during microscopic preparation; triple arrow, preserved crystals; white star, membranes inside a membranous chromoplast. *ld* lipid droplet, *sg* starch grain, *v* vacuole

Plastids in d-o callus cells differed in their ultrastructure, and chromoplasts of various types occurred often in the same cell. Crystalline chromoplasts were the most abundant (Table 1) and they were clearly distinguished by the presence of membranes resembling ribbon-like (Fig. 5a) or needle-like shapes (Fig. 5b). These preserved structures were the remnants of dissolved carotene crystals resulting from the use of a destructive preparation procedure for TEM; however, in some chromoplasts, unaffected crystals or their parts were still present (Fig. 5b). Globular chromoplasts had an ellipsoid shape and a few plastoglobuli enclosed inside the stroma. They were three times less frequently observed than crystalline chromoplasts (Table 1; Fig. 5c). Two other chromoplast types, membranous (Fig. 5d) and tubular (Fig. 5e) were infrequent. Membranous chromoplasts were characterized by several to over a dozen concentric internal double membranes (Fig. 5c and inset). Tubular chromoplasts were characterized by internal elements of an elongated tube-shaped appearance and aligned to bundles (Fig. 5e, inset 1). Transverse sections revealed that the outer tubule layer was electron dense and the inner part was electron translucent (Fig. 5e, inset 2). Very often undulated membranes located distant from the chromoplasts were detected in the cytoplasm (Fig. 5f). Such membrane shape indicates that carotene crystals could be very long, which was congruent with observations done using bright-field and polarized light microscopes. Phytoferritin crystals were occasionally detected in some chromoplasts (Fig. 5g). Moreover, large plastoglobuli were detected, and in some of them crystal remnants were present (Fig. 5g, inset).

### Unique pattern of carotenoid gene expression exists in orange callus cells

**Fig. 6:**
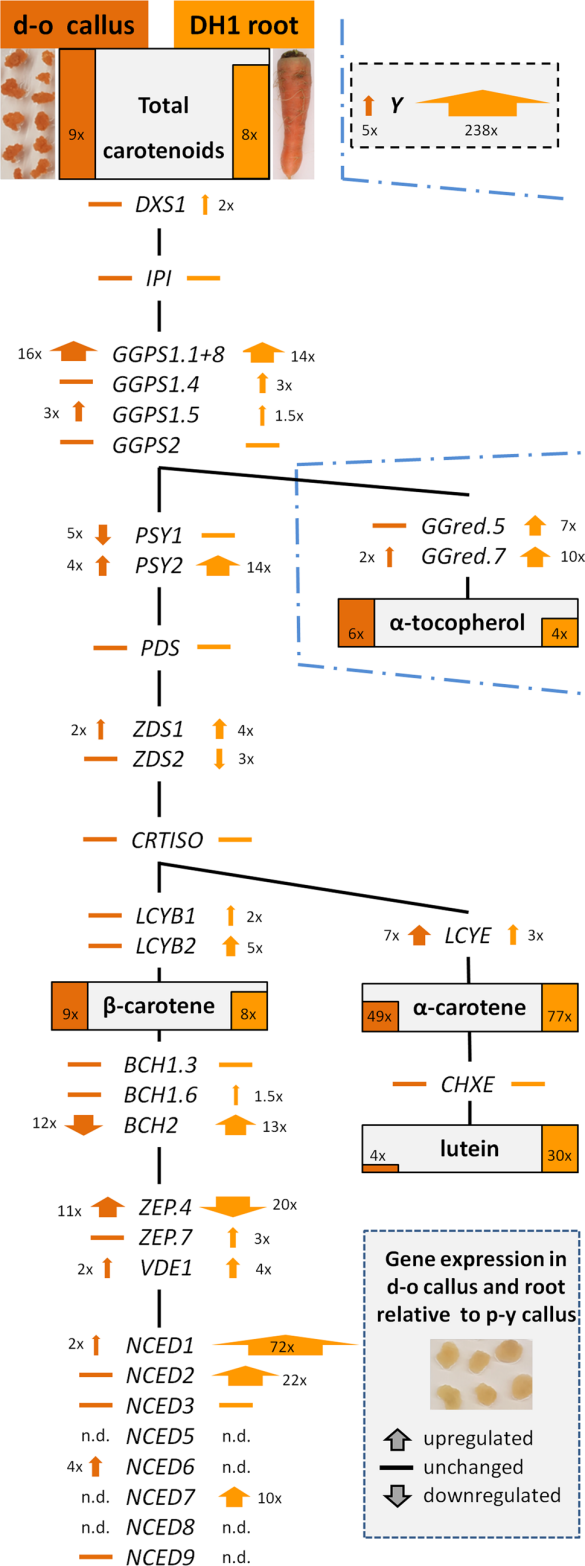
Schematic comparison of relative gene expression (to that in pale yellow callus) and carotenoid contents in dark orange (d-o) callus and roots. A simplified biosynthesis pathway of carotenoids and their precursors are shown with genes analyzed in this paper and listed in Suppl. Table S1. Carotenoid and α-tocopherol contents in d-o callus and roots are represented by dark orange and orange bars, respectively. The bar height is proportional to the relative content of a given compound in both tissues. Numbers show the compound content ratios relative to the contents in p-y callus. Gene expression levels, relative to the expression in pale yellow (p-y) callus, are represented by arrows with numbers indicating fold difference and arrow widths proportional to expression levels, or by a horizontal line if there is no significant change in expression level in comparison to p-y callus. Relative expression of the candidate regulatory *Y* gene is shown in the box with a dashed border. *n.d.* transcripts not detected. Exact expression values are given in Suppl. Table S2

Transcript levels of 33 genes associated with carotenoid biosynthesis (Electronic Suppl. Table S1) were measured using a quantitative RT-PCR approach in carotenoid-poor and carotenoid-rich callus and in the orange root which p-y callus was derived from, and differential expression was found among these materials. Genes involved in the biosynthesis of carotenoid precursors were expressed at similar levels in d-o callus and the roots, and *IPI* expression was similar in all three tissues, including p-y callus (Fig. 6; Electronic Suppl. Table S2). Three out five geranylgeranyl diphosphate (*GGPS*) paralogues were highly upregulated in carotenoid-rich tissues, i.e. *GGPS1.5* and particularly two genes located on chromosomes Ch1 (*GGPS1.1*) and Ch8 (*GGPS1.8*). The full nucleotide homology between *GGPS1.1* and *GGPS1.8* in the carrot genome did not allow separate quantification of their transcripts, whose levels were 14–16-fold higher in both tissues in comparison to the carotenoid-poor p-y callus.

The expression of most of 24 genes, including paralogues, of the main pathway for carotenoid biosynthesis and metabolism was much higher (2–72-fold) in the roots than in p-y callus (Fig. 6; Electronic Suppl. Table S2). Only two genes, ζ-carotene desaturase (*ZDS2*) and zeaxanthin epoxidase (*ZEP.4*) were downregulated in the roots, and transcript levels of five genes, phytoene synthase *PSY1*, phytoene desaturase (*PDS*), prolycopene isomerase (*CRTISO*), β-carotene hydroxylase *BCH1.3*, and carotene ε-monooxygenase (*CHXE*), were similar in both materials. The most downregulated genes in p-y callus were epoxycarotenoid dioxygenases (*NCED*s), *BCH2* and *PSY2*.

Both carotenoid-poor and carotenoid-rich callus lines had similar expression levels of 12 genes but the next nine genes had higher expression in d-o callus. Only two of them, *PSY2* and *ZDS1*, are involved at early steps of carotene biosynthesis and had 4.0-fold and 2.0-fold higher expression, respectively. Lycopene ε-cyclase (*LCYE*) and some xanthophyll cycle and *NCED* genes were upregulated in d-o callus. Two genes had lower expression in d-o callus than in p-y callus, i.e. *PSY1* (4.6-fold lower expression) and *BCH2* (11.9-fold), involved in β-carotene conversion to xanthophylls.

Notable differences in expression levels were observed between d-o callus and the roots despite both materials accumulating similar amounts of carotenoids. Transcripts of most genes in d-o callus were at the same or lower levels than in the roots, and the most prominent differences were observed for genes involved in carotenoid catabolism (*NCED*s), including *BCH2* (155-fold lower expression in d-o callus). The expression level of another paralogue, *BCH1.6*, was only slightly higher (1.6-fold) in the roots while expression of the *BCH1.3* gene was at a similar level in both tissues, apparently not different from that observed in p-y callus. Both *PSY1* and *PSY2* were differentially expressed in carotenoid-accumulating tissues, and their expression was about three-fold higher in the roots. *PSY1* expression in d-o callus was also lower than in p-y callus. In the α-branch carotenoid pathway, a higher *LCYE* transcript level was found in d-o callus than in both p-y callus and the roots, while *CHXE* expression was stable among all tissues.

A candidate gene (*Y*, DCAR_032551) regulating carotenoid accumulation in orange storage carrot root (Iorizzo et al. 2016) was also highly expressed in the root used in our study. Its transcript level was 238-fold and 49-fold lower in p-y callus and d-o callus, respectively.

Transcript levels of geranylgeranyl diphosphate reductase (*GGred*) variants involved in metabolite flux to the biosynthesis of phytyl chain compounds were seven-to-ten-fold lower in p-y callus, and 5–12-fold lower in d-o callus in comparison to the levels determined for root tissue.

## Discussion

Chromoplasts were rarely found in p-y callus cells, and thus only low amounts of carotenoids could be accumulated in this tissue. The small number of plastids and their simple organisation may explain the low carotenoid contents in carrot callus, also reported previously (Hanchinal et al. 2008). In contrast, cells of d-o callus contained high carotenoid amounts, notably being as high as in the carrot root. This carotenoid-rich d-o callus was distinguished from typical carrot callus at morphological, histological and ultrastructural levels, showing cell organisation common in embryogenic and meristematic tissues, and had a higher ABA content. Elevated ABA amounts were reported earlier in embryogenic calli derived from carrot hypocotyls, in contrast to non-embryogenic calli (Jimenez and Bangerth 2001). Thus, it was shown that cell organisation in the callus is related to the level of this plant hormone, which is a product of carotenoid catabolism. Due to its embryogenic character, d-o callus architecture was compact, so the increase of callus volume during the culture was slower than in the case of p-y callus, having larger cells and a loose structure. Higher ABA content could additionally explain slower d-o callus growth, as this hormone affects many physiological processes, including repressing the synthesis of cytokinin, whose limited availability in the tissue results in delayed cell division (Ha et al. 2012).

Chromoplasts are classified as four main types, i.e. crystalloid, globular, tubular and membranous, depending on their ultrastructure and chemical composition (Egea et al. 2010; Solymosi and Keresztes 2012; Schweiggert and Carle 2017). Usually one type of chromoplasts dominates in the tissue, but the coexistence of some other types, even in the same cell, is known. It is broadly accepted that the occurrence of different chromoplast types is a result of plastid differentiation and transition from one plastid type to another (Ljubesic et al. 1991; Pyke 2007). All four chromoplast types were observed in d-o callus, which is unusual and indicates highly complex plastid biogenesis occurring in this tissue, although crystalloid chromoplasts were the most abundant, unlike in p-y callus where they were hardly identified. It is well documented that in carrot root, crystalloid chromoplasts possess carotenoids in their crystalline form of different shapes (Frey-Wyssling and Schwegler 1965; Paolillo et al. 2004; Vasquez-Caicedo et al. 2006; Roman et al. 2015). When the concentration of carotenoids increases, they start to crystallize inside the chromoplast, and the growing crystals often heavily distort the shape of the chromoplast, which may extend to a large size (Li and Yuan 2013). Confirmation of crystalline carotenoid presence in TEM images is, however, indirect, as during sample fixation carotenoids dissolve and only some crystal remnants can be preserved and observed as electron-dense structures of regular shape. When carotenoids are completely washed out, chromoplast membranes surrounding the crystal collapse and are visible as characteristic undulating, electron-dense membrane spanning along the electron-lucent area void of the crystal (Kim et al. 2010). Crystalline structures have been frequently observed in d-o callus using light and polarizing microscopy, and partially using TEM. Both electron-dense and electron-lucent structures were clearly identified and often coexisted in one cell, indicating massive crystal accumulation. Recent spectroscopic measurements of crystals in d-o callus showed that they were composed predominantly of β-carotene accompanied by α-carotene, while co-occurrence of lutein was unlikely but their composition depended on the crystal structure (Rygula et al. 2018). Thus, measurements of carotenoid crystals in model d-o callus provided evidence supporting the previous hypothesis of heterogeneous composition of crystals accumulated in carrot root (Marx et al. 2003; Roman et al. 2015).

Other types of carotenoid-containing chromoplasts can be identified based on their ultrastructure visualized by TEM, and their classification is well presented (Schweiggert and Carle 2017). However, spectroscopic measurements using non-destructive approaches applied to intact cells provide additional support in carotenoid identification. It was revealed that carotenoid crystals and their lipoprotein complexes coexist in carrot root cells (Marx et al. 2003; Baranska et al. 2011; Roman et al. 2015). The presence of amorphous carotenoids, whose spectra indicate lipoprotein complexes, is congruent with observations of the non-crystalline chromoplasts in TEM images of d-o callus. Moreover, scanning near-field optical microscopy (SNOM) coupled with Raman spectroscopy applied directly to d-o callus cells revealed numerous, small and carotenoid-rich, membranous and tubular-like structures, which thus most likely were chromoplasts (Rygula et al. 2018), which is also congruent with the TEM results presented here.

Kim et al. (2010) observed the presence of globular chromoplasts in carrot root tissue, although their number was small. Globular chromoplasts contain numerous plastoglobuli and only fragments of membranes (Camara et al. 1995). Lipid-rich plastoglobuli are a suitable environment for the biosynthesis and deposition of many lipophilic constituents, and they are believed to have little function beyond lipid storage, although they are also considered as the most common carotenoid-containing structures in the chromoplasts (Schweiggert and Carle 2017). Cells of carrot d-o callus described in this paper also contained globular chromoplasts with electron-dense plastoglobuli and embedded small crystals visible in TEM images. The formation of carotene crystals inside plastoglobuli was also reported for tulip tree (*Liriodendron tulipifera*) flowers and squash (*Cucurbita maxima*) fruit (Ljubesic et al. 1991). As crystals in plastoglobuli in d-o callus were small, these structures are most likely involved in accumulation of soluble carotenoids and other lipophilic compounds, including tocopherols. Carotenoids stored within plastoglobuli exhibit much higher light stability than those associated with membranes, so plastoglobuli play an important role mainly in light-exposed plant organs (Vasquez-Caicedo et al. 2006). Thus, the smaller number of plastoglobuli found in d-o callus chromoplasts might result from tissue culture in the dark. Unexpectedly, membranous chromoplasts were identified in d-o callus cells. These plastids have a distinguished structure of multiple, usually up to 20, internal double membranes arranged concentrically (Schweiggert and Carle 2017). They are rarely found in plants and have been uniquely reported in some flowers and selected pepper and tomato cultivars (Sitte et al. 1980). Chromoplasts of the *Or* cauliflower (*Brassica oleracea* L. var. *botrytis*) mutant were classified as membranous (Paolillo et al. 2004). To our best knowledge, typical membranous chromoplasts have never been observed in carrot. Thus, the presence of membranous chromoplasts in carrot d-o callus is unique. Tubular chromoplasts were also observed in TEM images of d-o callus cells. They resembled a system of tubules of crystalline-like appearance and about 30 nm in diameter (see Fig. 5e) corresponds to a typical morphology known from other species where the average tube diameter range is 20–60 nm (Sitte et al. 1980). Tubular chromoplasts are bundles of elongated, sometimes branched, tubes that can reach up to 10 μm in length (Sitte et al. 1980) and have been found in many flowers and fruits (Camara et al. 1995; Schweiggert and Carle 2017). They are anisotropic structures with a birefringence property due to the presence of liquid-crystalline carotenoids, including β-carotene, and are sinks of lipid-dissolved carotenoid esters (Schweiggert et al. 2012; Hempel et al. 2017; Schweiggert and Carle 2017). The composition of tubes can be diverse, and lutein may constitute even a half of the lipophilic fraction, as was found in *Palisota barteri* (Knoth et al. 1986). The relatively low frequency of tubular chromoplasts in d-o callus may thus be related to low lutein content present in this tissue.

The presence of composed amylochromoplasts as well as typical crystalline, globular, membranous and tubular chromoplasts in d-o callus cells implies that carotenoid biosynthesis, sequestration and storage take place in chromoplasts of different ultrastructure. Such diversity supports the conclusion that chromoplast development can proceed in various ways (Solymosi and Keresztes 2012) and makes d-o callus a valuable model tissue for research on plastid biogenesis as it can be easily exposed to a range of stimuli. According to Kumar et al. (1984) amyloplasts may serve as chromoplast precursors; thus developing chromoplasts (amylochromoplasts) maintain small starch grains and can include globular and crystalline structures, making classification of chromoplasts ambiguous (Hempel et al. 2014). Amyloplasts were abundant in p-y callus while chromoplasts and amylochromoplasts were frequent in d-o callus. A large number of amylochromoplasts containing either plastoglobuli or crystalline structures, or both, indicates chromoplasts’ origin from amyloplasts in d-o callus. This route of chromoplast biogenesis has already been proposed to operate in carrot root. Amyloplasts were identified in young carrot roots but they disappeared during root development and were not observed in mature, carotenoid-rich, orange storage root. However, roots of white carrot, which are free of carotenoids, retained large amyloplasts (Kim et al. 2010). The authors hypothesized that the amyloplast-to-chromoplast transition occurred during carotenoid accumulation. The presence of amylochromoplasts in d-o callus cells supports this hypothesis and provides evidence of intermediate stages of chromoplast development that have not been demonstrated in carrot root so far, but were found in other species (Horner et al. 2007). The process of chromoplast development in d-o callus was also captured in ultrastructure images. Usually, the size of plastoglobuli ranges from 30 nm to more than 1000 nm (Sitte et al. 1980), although it depends on plastid type, species, organ and even environment (Brehelin et al. 2007), and the number of plastoglobuli increases with chromoplast maturation (Solymosi and Keresztes 2012). Thus, globular chromoplasts in d-o callus possessing a few small plastoglobuli seem to exemplify chromoplasts at their early stages of development. Chromoplast development can also be seen in the ultrastructure of membranous chromoplasts, which in d-o callus had a smaller number of concentric membrane layers than mature chromoplasts described in other species (Schweiggert and Carle 2017). This callus tissue also contained cells with ageing chromoplasts. The detection of large phytoferritin aggregates in some chromoplasts may be evidence of chromoplast senescence, which has not been reported in carrot root cells before. This conclusion is congruent with elevated ABA content in d-o callus that mediates senescence, including chlorophyll degradation and plastid degeneration, and non-toxic, protein–iron phytoferritin complexes present in the stroma are characteristic for ageing chromoplasts (Ljubesic 1982).

Several genes related to carotenogenesis were identified within the analysis of the carrot genome sequence (Iorizzo et al. 2016). A set of genes chosen for expression analyses in this work are primarily congruent with that list. It includes genes and their paralogues involved in biosynthesis of carotenoids and their precursors, and those involved in the main carotenoid degradation route through xanthophyll cycle carotenoids to ABA. Carotenoids may also be enzymatically degraded by carotenoid cleavage dioxygenases (CCDs), which affect carotenoid levels, as observed in *Arabidopsis* callus (Schaub et al. 2018). The existence of several *CCD* genes with paralogues in carrot makes their discrimination troublesome when using a qPCR approach; hence they were not included in this work. In the carrot genome, a region linked to the *Y* gene (DCAR_032551) associated with carotenoid accumulation has also been mapped recently, and its sequence analysis indicated homology to *Arabidopsis* PEL protein involved in photomorphogenesis. Only carrots possessing a mutant, putatively non-functional PEL accumulated carotenoids (Iorizzo et al. 2016). The authors hypothesized that the *yy* mutant plant was not able to repress photomorphogenesis, and thus plastids, either chloroplasts in the light or chromoplasts in the dark, can develop, synthesize, and sequester carotenoids. The hypothesis was supported by transcriptome analysis showing coordinated expression of other genes, such as COP1 and HY5 cytochrome-associated proteins, known to be involved in photomorphogenesis, and interacting with PSY expression (Llorente et al. 2017). We used the same genetic material, i.e. a homozygous recessive *yy* mutant as described by Iorizzo et al. (2016). The relative expression of the *Y* (DCAR_032551) gene in d-o callus was almost five-fold higher than in p-y callus and over 50-fold lower than in the root of the growing DH1 plant. The higher expression in d-o callus than in p-y callus partially corresponds to the higher carotenoid level. However, the much more pronounced lower expression in d-o callus than in the root, containing similar carotenoid amounts, indicates that accumulation of carotenoids is unlikely to be related to the differences in DCAR_032551 expression. These results are not contradictory to the above-mentioned hypothesized role of DCAR_032551 assuming this gene codes for a non-functional protein, although conclusions based on the comparison of the callus model system to the growing plant must be drawn with caution.

Gene upregulation in the upstream pathway causing carotenoid precursors’ flux is essential for downstream metabolite biosynthesis and in consequence their accumulation (Rodriguez-Concepcion 2010). One of the most prominent differences in transcript levels between both carotenoid-rich d-o callus and roots, and carotenoid-poor p-y callus was identified for *GGPS*. The transcript levels of mainly *GGPS1* present in two copies in the carrot genome were essentially similar in both carotenoid-rich d-o callus and roots, and about 15-fold higher than in carotenoid-poor p-y callus. Thus, their high expression most likely enhanced biosynthesis of geranylgeranyl diphosphate molecules, which are substrates for PSY, the first enzyme in the carotenoid pathway. Usually, PSY is the major rate-limiting factor of carotenoid biosynthesis in many organisms (Giuliano 2014). The key role of this gene was shown in non-green *Arabidopsis* tissues, including callus. The overexpression of the *AtPSY* gene resulted in an increased PSY protein level and in consequence carotenoid contents, although the expression of other carotenoid genes remained unaffected. Callus accumulated high β-carotene amounts deposited also as crystals that resembled sequestration as observed in carrot root (Maass et al. 2009). The differential role of PSY isoforms depending on the plant organ was also shown with plastid localization associated mainly with plastoglobuli (Shumskaya and Wurtzel 2013). Two *PSY* genes, *PSY1* and *PSY2*, have been described in carrot, and their expression has been reported (Maass et al. 2009; Iorizzo et al. 2016). Additionally, a partial *PSY2* sequence was predicted after genome sequencing, also known as *PSY3* in the NCBI nucleotide database, but it has a low homology to *PSY3* genes in other species and there is no confirmation of its functional role, so it was not included in our investigation. PSY1 seems more important in green tissues than in the root as *PSY1* expression is upregulated in leaves (Wang et al. 2014). Moreover, it responds to light regulation and becomes active in de-etiolated root tissues. The expression of *PSY2* in the root remains unaffected by light while it is repressed in leaves (Fuentes et al. 2012; Wang et al. 2014). However, Maass et al. (2009) showed that the *PSY1* transcript level was two-to-three-fold higher than *PSY2* in cultivars with white and orange roots. In our work, *PSY1* was downregulated in the dark-grown d-o callus in comparison to p-y callus, and thus its expression could not contribute to the increase of carotenoid level in d-o callus. In contrast, comparing both callus lines, *PSY2* was upregulated in orange tissue, which corresponded to enhanced phytoene biosynthesis. The increased *PSY2* expression in the dark indicates different activity of *PSY1* and *PSY2* genes depending on the tissue. This corroborates differential *PSY* gene expression found in carrot root phloem tissue containing higher carotenoid contents than xylem. The expression of *PSY2* in phloem was much higher relative to xylem, while *PSY1* was stable or only slightly increased (Perrin et al. 2017). However, high carotenoid accumulation in d-o callus can be only partially explained by *PSY2* upregulation, as its transcript level was almost four-fold lower than in the root, containing similar amounts of carotenoids. This is congruent with the incomplete correlation found between *PSY* transcript levels and carotenoid contents in white and orange roots (Bowman et al. 2014). Moreover, reduced PSY enzyme levels did not correspond to unchanged PSY transcript levels, indicating that the protein level is modulated by carotenoid metabolites (Arango et al. 2014). Recent application of another callus-based system in *Arabidopsis* indicated that low carotenoid contents in callus may result from non-enzymatic carotene degradation, which was compensated by only high PSY overexpression enabling carotenoid accumulation (Schaub et al. 2018).

Conversion of cyclic carotenes to xanthophylls is mediated by hydroxylases and BCH is the main enzyme hydroxylating β-carotene (Moise et al. 2014); thus the repression of BCH may also result in higher β-carotene accumulation (Giuliano 2014). The *BCH1* expression levels were similar in both callus tissues and in the roots, while the *BCH2* expression was highly repressed in d-o callus compared to the root, and its level was additionally 12-fold lower than in carotenoid-poor p-y callus. These results indicate that β-carotene accumulated in d-o callus mainly as the result of repressed β-carotene hydroxylation due to *BCH2* downregulation. This mechanism is thus analogous to that observed at a late stage of carrot root development where after 12 weeks of vegetation the *BCH2* expression was highly repressed in the dark-grown roots, preventing β-carotene catabolism and contributing to carotenoid accumulation (Fuentes et al. 2012).

Interestingly, the amounts of α-branch carotenoids in d-o callus were lower than in the root despite higher LCYE expression in callus. Two enzymes, lycopene β-cyclase (LCYB) and LCYE, responsible for lycopene cyclisation compete for the substrate. LCYB is responsible for lycopene to β-carotene conversion, while both LCYB and LCYE are involved in the biosynthesis of α-carotene possessing one β- and one ε-ionone ring (Moise et al. 2014). Thus, relative activities of both enzymes affect β-carotene to α-carotene ratio (Giorio et al. 2013). It was shown that LCYE overexpression led to higher lutein accumulation in tomato (Giorio et al. 2013) while its downregulation elevated β-carotene amounts in potato, channelling the substrate to the β-branch pathway (Diretto et al. 2007; Kim et al. 2013). A significant increase of *LCYE* expression was also observed during carrot root maturation, which partially explained the increase of α-branch carotenoids (Fuentes et al. 2012). The results presented here show that *LCYB* genes were expressed at the same level in carotenoid-poor and carotenoid-rich callus lines, but the *LCYE* transcript level was significantly increased in d-o callus, notably also in relation to the roots. Nevertheless, α-carotene level was lower than in the roots. As LCYE may also exhibit activity for β-ionone ring formation (Bai et al. 2009) it could partially contribute to the enhanced β-carotene biosynthesis, making α-carotene biosynthesis less effective.

Lower α-carotene availability may only partially explain low lutein content in the callus. Hydroxylation of α-carotene at both β- and ε-ionone rings is required for lutein biosynthesis, and the conversion is preferentially catalyzed by CYP97 haem-containing cytochrome P450 hydroxylases (Kim and DellaPenna 2006; Moise et al. 2014). It was also shown in rice that interaction of CYP97A4 and CYP97C2 is required for efficient hydroxylation of both β- and ε-ionone rings, respectively (Quinlan et al. 2012). The carrot CHXE amino acid sequence shows homology to CYP97C enzyme family (Rodriguez-Concepcion and Stange 2013), while a mutant *CYP97A3* gene coding for a non-functional hydroxylase was identified in developing orange roots, and its presence correlated with high α-carotene content due to restricted α-carotene to lutein conversion (Arango et al. 2014). As CYP97A3 is not functional in carrot, most likely lutein biosynthesis in carrot requires coordinated activity of BCH and CHXE. However, in d-o callus the *BCH2* expression was highly repressed, which could not only enhance β-carotene accumulation but simultaneously restrict α-carotene hydroxylation at the β-ring, hence limiting lutein biosynthesis.

The results presented here indicate that two callus lines developed from the same orange carrot root have contrasting potential for carotenogenesis that coincides with differential expression of some genes associated with carotenoid biosynthesis and metabolism. A differential role of gene paralogues was found in a high-carotene, orange callus. This tissue had a pronounced cell organisation with abundant crystalline chromoplasts common also in carrot root. Additionally, membranous chromoplasts, rarely occurring in plants, accompanied globular and tubular chromoplasts, and amylochromoplasts. Hence, this orange callus accumulating similar amounts of carotenoids as carrot root is a unique model tissue possessing chromoplasts representing all known ultrastructural types, whose co-occurrence has not been observed in carrot root tissue until now. Carrot callus can be conveniently maintained in laboratory conditions, so the d-o callus described here can be highly advantageous for elucidating both chromoplast biogenesis and associated carotenogenesis when it is exposed to a range of environmental factors. Moreover, it can be easily genetically manipulated and may serve as a valuable material for implementing CRISPR technology enabling precise, targeted genome editing aimed at elucidating the role of carotenogenesis-associated genes.

### *Author contributions statement*

TO, MK-Ch and RB designed the research; TO performed callus culture in vitro; the following authors performed, analyzed and interpreted results: TO, AM-H and EK: light microscopy; AM-H and EK: polarizing microscopy and histology; AM-H, MZ, DS and EK: transmission electron microscopy; AB and JS: UPLC; TO and MK-Ch: bioinformatics and RT-qPCR; RB interpreted results and coordinated work; TO, MK-Ch, AM-H, EK and RB wrote the manuscript.

## Electronic supplementary material

Below is the link to the electronic supplementary material.

Supplementary material 1 (XLSX 15 kb)

Supplementary material 2 (XLSX 13 kb)

## Abbreviations

d-o: Dark orange
p-y: Pale yellow
PSY: Phytoene synthase
